# Economic evaluations  of strategies targeting pre-diagnosis dementia  populations: Protocol for a systematic review

**DOI:** 10.12688/hrbopenres.14064.1

**Published:** 2025-01-23

**Authors:** Men Thi Hoang, Alina Zenker, Sanjib Saha, Ulf-Göran Gerdtham, Dominic Trepel

**Affiliations:** 1 Trinity College Dublin, Trinity College Institute of Neuroscience, Dublin, Leinster, Ireland; 2 Trinity College Dublin School of Medicine, Dublin, Leinster, Ireland; 3 Faculty of Business Administration, Van Lang University, Ho Chi Minh City, Vietnam; 4 Health Economics Unit, Department of Clinical Science (Malmö), Lund University, Lund, Sweden; 5 Department of Economics, Lund University, Lund, Sweden; 6 Global Brain Health Institute, Trinity College Dublin, Dublin, Ireland

**Keywords:** Cost-effectiveness Analysis, economic evaluation, dementia, Alzheimer's Disease, identification, treatment

## Abstract

**Introduction:**

Dementia remains incurable, and treatment trials are typically conducted after the symptoms manifest, potentially too late in the disease process to alter its course. Early identification and intervention during the pre-diagnosis phase offer the potential to introduce more cost-effective strategies and enhance quality of life. This review aims to scrutinise emerging evidence and present a comprehensive summary of cost-effectiveness estimates of all strategies targeting the pre-diagnosis dementia population.

**Method and analysis:**

A systematic search will be conducted across six electronic databases. All articles will be assessed against pre-defined eligibility criteria through title and abstract screening, and full-text screening phases. Data from the included articles will be extracted using a standardized template. A newly established framework based on the CHEERS 2022 checklist will be applied to assess the reporting quality of the included articles. The entire review process, from screening to data extraction and quality assessment, will be a dual process conducted by two reviewers. Disagreements will be resolved by a third senior reviewer. The extracted data will be synthesised and presented in tables and figures.

**Conclusion:**

This systematic review will present evidence of cost-effectiveness, along with the strengths and limitations of the existing literature. These findings aim to identify existing gaps, thereby informing and guiding the design of future studies in this domain.

**Ethics and dissemination:**

Since this is a systematic review protocol, ethical approval is not required. The results will be published in a peer-reviewed journal, with both raw and summarised data shared through the journal or other open platforms.

**Systematic review registration:**

PROSPERO - CRD42024521521.

## Introduction

Dementia is a progressive condition characterised by deterioration of multiple cognitive domains that significantly affects daily functioning. According to the World Health Organization (WHO) over 55 million people worldwide live with dementia, with approximately 10 million new cases emerging annually ^1^. It is the seventh leading cause of death and contributes to disability and dependency, affecting individuals, caregivers, and society ^1^.

Currently, there is no cure for dementia, and treatment trials are typically conducted after symptoms manifest, often too late to alter disease progression ^2^. Early identification and intervention during the pre-diagnosis phase of dementia offer the potential to introduce effective strategies aimed at slowing or halting disease progression, commonly referred to as disease-modifying treatments (DMTs), or modifying risk factors that may effectively prevent or delay dementia ^3^. Moreover, considering the high costs associated with long-term care, early strategies, such as screening tests that enable individuals to maintain independent living and extend the duration of residence with their families in communities, are deemed to be "cost-effective" and may enhance the quality of life ^4^.

A systematic review is well suited to examine common characteristics and evaluate the quality of existing literature, aiding in the identification of research gaps and guiding future studies. Previous systematic reviews on the economic evaluation (EE) of dementia have predominantly focused on specific strategies or categories for mild cognitive impairment (MCI) and dementia ^5– 7^, Alzheimer’s disease ^8, 9^ or the examination of methodological techniques employed in EEs, such as model-based EE ^10, 11^. However, a comprehensive review encompassing all strategies specifically targeting pre-diagnosis dementia is lacking in the literature. Furthermore, most existing reviews are outdated and were conducted between 2008 and 2017 ^6– 11^. This systematic review aimed to address these gaps by analysing emerging evidence and providing a comprehensive summary of EEs for strategies targeting the pre-diagnosis dementia population.

## Method

### Registration

This systematic review protocol follows the Preferred Reporting Items for Systematic Reviews and Meta-Analyses Protocols (PRISMA-P) guidelines ^12^. The PRISMA-P checklist is provided as part of the Extended Data ^13, 14^. The review was registered with the International Prospective Register of Systematic Reviews (PROSPERO CRD42024521521). The results will be reported in accordance with the Preferred Reporting Items for Systematic Reviews and Meta-Analyses (PRISMA) guidelines ^15^.

### Eligibility criteria

*Population:* The target population includes individuals at any stage prior to having a formal diagnosis of dementia, such as the general population, people at risk of dementia, those with mild cognitive impairment (MCI), caregivers, and patient-caregiver dyads. Pre-diagnosis populations are commonly pre-defined based on either a Mini-Mental State Examination (MMSE) score equal to >=24 or explicitly labelled as “no dementia” cohorts or MCI populations.

*Intervention:* All types of strategies that target our pre-specified pre-diagnosis dementia population will be included. As a result, four categories of intervention will be included: (1) identification, (2) pharmacotherapy, (3) non-pharmacotherapy, and (4) management strategies at home, in the community, and in long-term care.

Articles will only be included if the strategies under investigation specifically target the aforementioned populations.

*Comparator:* The comparison group will contain any of the following: (1) usual care or standard of care or conventional interventions, (2) placebo, (3) doing nothing or without intervention, or (4) any treatment aimed at a pre-defined population.

*Outcomes:* Our primary outcomes will be cost-effectiveness assessments, such as incremental cost-effectiveness ratio (ICER), net monetary benefit (NMB), net health benefit (NHB), costs, incremental cost, effectiveness outcomes such as quality-adjusted life-year (QALY), and incremental effectiveness outcomes (e.g., incremental QALY). Additional outcomes will be included, such as clinical outcomes, which were used to indicate cost-effectiveness, and data related to resource use and utility.

*Study design:* Full EEs will be selected for this review. Full EEs were pre-defined according to Drummond’s classification scheme ^16^. Accordingly, this review will include (1) Cost-Effectiveness Analysis (CEA), cost-utility analysis (CUA), and Cost-Benefit Analysis (CBA).

*Settings:* The review includes studies conducted in all countries globally.

### Exclusion criteria

Articles will be excluded from the review if they (1) do not target pre-diagnosis dementia, or populations with unclear boundaries surrounding diagnosis (i.e., some pre- and some post-diagnosis) or individuals who may only exhibit MCI symptomatology but where neuropathology of a dementia has been established (e.g., MCI due to Alzheimer’s Disease (AD)), (2) partial EEs such as cost-of-illness analysis, efficacy effectiveness evaluation, cost-outcome description, (3) reviews, editorials, letters, commentaries, conference abstracts, (4) proceedings, protocols, case studies, and (5) are not reported in English.

### Search strategy

Potentially relevant literature was identified by systematically searching six electronic databases: Embase, PubMed, Econlit, Web of Science, Cumulative Index to Nursing and Allied Health (CINAHL) and The NHS Economic Evaluation Database (NHS EED). The details of the search terms are provided in Extended Data ^13, 14^.

### Study selection

References exported from the six databases will be transferred to EndNote, deduplicated, and then imported into the Covidence ( https://www.covidence.org).

The study selection process will be conducted independently by two reviewers. Any disagreement will be resolved by a third reviewer. During the screening process, all titles and abstracts will be independently scanned for relevance, and the full texts of eligible articles will be assessed according to pre-defined inclusion and exclusion criteria.

### Data extraction

The data will be extracted into a pre-defined template using Covidence. This process will be conducted independently by two reviewers, and any disagreements will be resolved by a third reviewer.

The extracted items will include:

*Study details*: Title, author, publication information, country, funding source, study design, population, intervention, comparator, and settings.

*Methodology*: Type of EEs, type of model (if applicable), time horizon, perspective, type of sensitivity analysis, currency, price, discount rate, method to measure and value costs, and method to measure and value outcomes.

*Results*: Total costs, incremental cost, total outcomes, incremental outcomes, ICER, NMB, and authors’ conclusions on the cost-effectiveness of the strategies.

### Data synthesis

Pre-diagnosis dementia strategies will be categorized into four groups: identification, pharmacological and non-pharmacological interventions, and management.

Qualitative synthesis will report the number of EE studies in each subgroup. Where studies report both total costs and outcomes expressed in QALYs, results will be adjusted and will be visually presented on a cost-effectiveness plane to facilitate decision-making.

According to the available data and the variation in study quality, method, or design, narrative synthesis provides guidance on the interpretation and reliability of estimates to inform allocation decisions. The extracted data will be presented in a summary table of the key extracted items. Figures will be used to portray the production of studies over time and geographic location.

### Quality appraisal

The Consolidated Health Economic Evaluation Reporting Standards (CHEERS) 2022 checklist was initially piloted to evaluate the reporting quality ^17^. However, discrepancies arose during this process owing to varying interpretations by the two reviewers, leading to inconsistencies across items. To address these issues, several meetings were held with senior reviewers (DT and SS), resulting in a modified CHEERS 2022 framework comprising 67 sub-items, categorized as follows: title (two items), abstract (four items), introduction (three items), methods (38 items), results (11 items), discussion (six items), and other relevant information (two items). This adapted framework will be applied to assess reporting quality. Two reviewers will independently assess the reporting quality of the included articles using this framework, with a third reviewer resolving disagreements. All processes will be performed using Covidence.

Results of the reporting quality of included articles will be summarised using the traffic light system (i.e., green: fully satisfied, amber: partially satisfied, or red: where the standard was not achieved). Scoring of individual studies, in line with the framework, will be summarised in the summaries of each paper.

## Discussion

EE is a useful analytical technique for informing policy decisions on resource allocation by providing cost-effective evidence for strategies. This systematic review will be the first to specifically target pre-diagnosis dementia populations, addressing a significant gap in the literature.

This review includes both trial-based and model-based EEs. It will not only focus on cost-effectiveness outcomes but also on methodology, data sources, and methods for measuring and valuing the costs and outcomes of existing EEs. Narrative synthesis guides the interpretation and reliability of estimates to inform allocation decision making. The results for incremental costs and incremental QALYs will be displayed on a cost-effectiveness plane when data permits, facilitating decision making.

Additionally, this systematic review will introduce the utilisation of a newly established framework adapted from CHEERS 2022 to assess the reporting quality of the included articles. Reporting quality assessment will be summarised using the traffic light system to visualise existing problems in reporting the quality of existing EEs.

This systematic review will summarise cost-effectiveness evidence, highlighting the strengths and limitations of the current literature. The findings will help identify research gaps, thereby improving the design and development of future research.

## Data Availability

No data are associated with this article. Figshare: Extended data file - Economic evaluations of strategies targeting pre-diagnosis dementia populations: Protocol for a Systematic Review, https://doi.org/10.6084/m9.figshare.28235993 ^13^. This project contains the following extended data: PRISMA-P Checklist Search strategies Figshare: PRISMA-P checklist for ‘Economic evaluations of strategies targeting pre-diagnosis dementia populations: Protocol for a Systematic Review’, https://doi.org/10.6084/m9.figshare.28235993 ^13^. Data are available under the terms of the Creative Commons Attribution 4.0 International license (CC-BY 4.0).
