# Economic evaluations  of strategies targeting pre-diagnosis dementia  populations: Protocol for a systematic review

**DOI:** 10.12688/hrbopenres.14064.3

**Published:** 2025-09-29

**Authors:** Men Thi Hoang, Alina Zenker, Sanjib Saha, Ulf-Göran Gerdtham, Dominic Trepel

**Affiliations:** 1 Trinity College Institute of Neuroscience, Trinity College Dublin, Dublin, Leinster, Ireland; 2 School of Medicine, Trinity College Dublin, Dublin, Leinster, Ireland; 3 Faculty of Business Administration, Van Lang University, Ho Chi Minh City, Vietnam; 4 Health Economics Unit, Department of Clinical Science (Malmö), Lund University, Lund, Sweden; 5 Department of Economics, Lund University, Lund, Sweden; 6 Global Brain Health Institute, Trinity College Dublin, Dublin, Ireland

**Keywords:** Cost-effectiveness Analysis, economic evaluation, dementia, Alzheimer's Disease, identification, treatment

## Abstract

**Introduction:**

Dementia remains incurable, and treatment trials are typically conducted after the symptoms manifest, potentially too late in the disease process to alter its course. Thus, it is essential to identify and implement cost-effective strategies targeting individuals who have not yet been formally diagnosed with dementia. This review aims to scrutinise emerging evidence and present a comprehensive summary of cost-effectiveness estimates of all strategies targeting the pre-diagnosis dementia populations.

**Method and analysis:**

A systematic search will be conducted across six electronic databases. All articles will be assessed against pre-defined eligibility criteria through title and abstract screening, and full-text screening phases. Data from the included articles will be extracted using a standardized template. A newly established framework based on the CHEERS 2022 checklist will be applied to assess the reporting quality of the included articles. The entire review process, from screening to data extraction and quality assessment, will be a dual process conducted by two reviewers. Disagreements will be resolved by a third senior reviewer. The extracted data will be synthesised and presented in tables and figures.

**Conclusion:**

This systematic review will present evidence of cost-effectiveness, along with the strengths and limitations of the existing literature. These findings aim to identify existing gaps, thereby informing and guiding the design of future studies in this domain.

**Ethics and dissemination:**

Since this is a systematic review protocol, ethical approval is not required. The results will be published in a peer-reviewed journal, with both raw and summarised data shared through the journal or other open platforms.

**Systematic review registration:**

PROSPERO - CRD42024521521.

## Introduction

Dementia is a group of conditions characterised by deterioration of multiple cognitive domains that significantly affects daily functioning. According to the World Health Organization (WHO) over 55 million people worldwide live with dementia, with approximately 10 million new cases emerging annually ^1^. It is the seventh leading cause of death and contributes to disability and dependency, affecting individuals, caregivers, and society ^1^.

Currently, there is no cure for dementia, and treatment trials are typically conducted after symptoms manifest, often too late to alter disease progression ^2^. Early identification of dementia is crucial for implementing effective treatment and care strategies; however, achieving this remains elusive due to the high prevalence of undiagnosed cases. In high-income countries, the diagnosis rate is approximately 42%, while globally, up to 75% of dementia cases may remain undiagnosed ^3, 4^. Thus, it is essential to identify and implement cost-effective strategies targeting individuals who have not yet been formally diagnosed (i.e., those in the pre-diagnosis dementia populations) to help reduce the rising impact of dementia.

A systematic review is well-suited to examine common characteristics and evaluate the quality of existing literature, thereby aiding in the identification of research gaps and guiding future studies. Previous systematic reviews on the economic evaluation (EE) of dementia have predominantly focused on specific strategies or categories for mild cognitive impairment (MCI) and dementia ^5– 7^, Alzheimer’s disease ^8, 9^ or the examination of methodological techniques employed in EEs, such as model-based EE ^10, 11^. However, a comprehensive review encompassing all strategies specifically targeting pre-diagnosis dementia populations, which include the general population, at-risk groups, and individuals with MCI without detectable AD pathology, is lacking in the literature. Furthermore, most existing reviews are outdated and were conducted between 2008 and 2017 ^6– 11^. This systematic review aimed to address these gaps by critically identifying evidence and providing a comprehensive summary of EEs for strategies targeting the pre-diagnosis dementia population.

## Method

### Registration

This systematic review protocol follows the Preferred Reporting Items for Systematic Reviews and Meta-Analyses Protocols (PRISMA-P) guidelines ^12^. The PRISMA-P checklist is provided as part of the Extended Data ^13^. The review was registered with the International Prospective Register of Systematic Reviews (PROSPERO CRD42024521521). The results will be reported in accordance with the Preferred Reporting Items for Systematic Reviews and Meta-Analyses (PRISMA) guidelines ^14^.

### Eligibility criteria

*Population:* The target population includes individuals at any stage prior to having a formal diagnosis of dementia, such as the general population, people at risk of dementia, those with mild cognitive impairment (MCI), caregivers, and patient-caregiver dyads. The pre-diagnosis dementia population is typically defined by authors who explicitly state that their studies start with a cohort described as having “no dementia,” or as comprising individuals with mild cognitive impairment (MCI) or at-risk population.

*Intervention:* All strategies targeting individuals in the pre-diagnosis phase of dementia will be considered. To be eligible, interventions needed to demonstrate an intended impact prior to the formal confirmation of dementia. The strategies encompassed: (1) Strategies for identifying people at risk of developing dementia; (2) strategies aimed at modifiable risk factors (such as exercise, nutrition, or cognitive training); and (3) Management-related initiatives, including models of healthcare delivery applied before a dementia diagnosis. Inclusion required that the intervention influence outcomes during the pre-diagnostic period, rather than solely detecting undiagnosed cases for later treatment.

*Comparator:* The comparison group will contain any of the following: (1) usual care or standard of care or conventional interventions, (2) placebo, (3) doing nothing or without intervention, or (4) any treatment aimed at the pre-defined populations.

*Outcomes:* Our primary outcomes will be cost-effectiveness assessments, such as incremental cost-effectiveness ratio (ICER), total costs, intervention costs, incremental cost, total effectiveness such as quality-adjusted life-year (QALY), and incremental effectiveness (e.g., incremental QALY).

*Study design:* Full EEs will be selected for this review. Full EEs were pre-defined according to Drummond’s classification scheme ^15^. Accordingly, this review will include (1) Cost-Effectiveness Analysis (CEA), cost-utility analysis (CUA), and Cost-Benefit Analysis (CBA), and Cost-Consequence Analysis (CCA).

*Settings:* The review includes studies conducted in all countries globally, and includes all types of clinical settings.

### Exclusion criteria

Articles will be excluded from the review if their:

Populations: do not target pre-diagnosis dementia, or populations with unclear boundaries surrounding diagnosis (i.e., some pre- and some post-diagnosis) or individuals who may only exhibit MCI symptomatology but where neuropathology of dementia has been established (i.e., MCI due to Alzheimer’s Disease (AD)). This is because the criteria for MCI due to AD must be confirmed by biomarkers to determine the likelihood that the syndrome results from the underlying pathophysiology of AD ^16^.

Interventions: (1) do not specifically target the defined pre-diagnosis populations; (2) the interventions solely detect individuals without a dementia diagnosis and provided risk-reduction or management strategies afterward, meaning they do not generate any impact during the pre-diagnostic phase; (3) studies encompass both the pre-diagnosis and post-diagnosis phases within the same design (for instance, integrating early detection with treatment for individuals already diagnosed). Such studies will be examined in a separate review.

Comparator: do not include any comparator.

Outcomes: do not report economic outcomes.

Study design: (1) partial EEs such as cost-of-illness analysis, efficacy effectiveness evaluation, cost-outcome description; (2) reviews, editorials, letters, commentaries, viewpoint/perspective, conference abstracts; (3) proceedings, protocols, case studies, and (4) are not reported in English.

### Search strategy

Potentially relevant literature was identified by systematically searching six electronic databases: Embase, PubMed, Econlit, Web of Science, Cumulative Index to Nursing and Allied Health (CINAHL) and The NHS Economic Evaluation Database (NHS EED). We searched for articles published between January 1, 2000, and January 14, 2025. The details of the search terms are provided in Extended Data ^13^.

### Study selection

References exported from the six databases will be transferred to EndNote, deduplicated, and then imported into the Covidence ( https://www.covidence.org).

The study selection process will be conducted independently by two reviewers. Any disagreement will be resolved by a third reviewer. During the screening process, all titles and abstracts will be independently scanned for relevance, and the full texts of eligible articles will be assessed according to pre-defined inclusion and exclusion criteria.

### Data extraction

The data will be extracted into a pre-defined template using Covidence. This process will be conducted independently by two reviewers, and any disagreements will be resolved by a third reviewer.

The extracted items will include:

*Study details*: Title, author, publication information, country, funding source, study design, population, intervention, comparator, and settings.

*Methodology*: Type of EEs, type of model (if applicable), time horizon, perspective, type of sensitivity analysis, currency, price, discount rate, method to measure and value costs, and method to measure and value outcomes.

*Results*: Total costs, incremental cost, total outcomes, incremental outcomes, ICER, NMB, and authors’ conclusions on the cost-effectiveness of the strategies.

### Data synthesis

Pre-diagnosis dementia strategies will be categorised into three groups: identification, risk reduction, and others.

Qualitative synthesis will report the number of EE studies in each subgroup. Where studies report both incremental costs and incremental outcomes expressed in QALYs, the incremental cost will be converted to international dollars (value in 2024) using the purchasing power parity from the International Monetary Fund dataset ^17^, and visually presented on a cost-effectiveness plane to facilitate comparison. Two willingness-to-pay (WTP) benchmarks were used to demonstrate the comparative cost-effectiveness of the strategies: $150,000 per QALY, indicative of the relatively unregulated market context in the United States (US) ^18^, and £12,936 per QALY, representing the more resource-limited system in the United Kingdom (UK) ^19^. These values were selected to capture the range between higher and lower reference points. The cost-effectiveness plane is used solely for illustrative purposes, we will not alter the original conclusion regarding the cost-effectiveness of the included articles.

According to the available data and the variation in study quality, method, or design, narrative synthesis provides guidance on the interpretation and reliability of estimates to inform allocation decisions. The extracted data will be presented in a summary table of the key extracted items. Figures will be used to portray the production of studies over time and geographic location.

All systematic review processes aim to adhere to standards set out in the Cochrane Handbook and specifically Chapter 20: Economic evidence ^20^. The results are reported in accordance with the Preferred Reporting Items for Systematic Reviews and Meta-Analyses (PRISMA) guidelines ^14^. The template for summarising data extracted from each individual study was based on the former template of CRD NHS EED database.

### Quality appraisal

The Consolidated Health Economic Evaluation Reporting Standards (CHEERS) 2022 checklist was initially piloted to evaluate the reporting quality ^21^. However, discrepancies arose during this process owing to varying interpretations by the two reviewers, leading to inconsistencies across items. To address these issues, several meetings were held with senior reviewers (DT and SS), resulting in a modified CHEERS 2022 framework comprising 67 sub-items, categorized as follows: title (two items), abstract (four items), introduction (three items), methods (38 items), results (11 items), discussion (six items), and other relevant information (two items). This adapted framework will be applied to assess reporting quality. Two reviewers will independently assess the reporting quality of the included articles using this framework, with a third reviewer resolving disagreements. All processes will be performed using Covidence.

Results of the reporting quality of included articles will be summarised using the traffic light system (i.e., green: fully satisfied, amber: partially satisfied, or red: where the standard was not achieved). Scoring of individual studies, in line with the framework, will be summarised in the summaries of each paper.

## Discussion

EE is a useful analytical technique for informing policy decisions on resource allocation by providing cost-effective evidence for strategies. This systematic review will be the first to specifically target pre-diagnosis dementia populations, addressing a significant gap in the literature.

This review includes both trial-based and model-based EEs. It will not only focus on cost-effectiveness outcomes but also on methodology, data sources, and methods for measuring and valuing the costs and outcomes of existing EEs. Narrative synthesis guides the interpretation and reliability of estimates to inform allocation decision making. The results for incremental costs and incremental QALYs will be displayed on a cost-effectiveness plane when data permits, facilitating decision making.

Additionally, this systematic review will introduce the utilisation of a newly established framework adapted from CHEERS 2022 to assess the reporting quality of the included articles. Reporting quality assessment will be summarised using the traffic light system to visualise existing problems in reporting the quality of existing EEs.

This systematic review will summarise cost-effectiveness evidence, highlighting the strengths and limitations of the current literature. The findings will help identify research gaps, thereby improving the design and development of future research.

## Data Availability

No data are associated with this article. Figshare: Extended data file - Economic evaluations of strategies targeting pre-diagnosis dementia populations: Protocol for a Systematic Review, https://doi.org/10.6084/m9.figshare.28235993.v2 ^13^. This project contains the following extended data: PRISMA-P Checklist Search strategies Figshare: PRISMA-P checklist for ‘Economic evaluations of strategies targeting pre-diagnosis dementia populations: Protocol for a Systematic Review’, https://doi.org/10.6084/m9.figshare.28235993.v2 ^13^. Data are available under the terms of the Creative Commons Attribution 4.0 International license (CC-BY 4.0).
