# Supplementary material for: Economic evaluations  of strategies targeting pre-diagnosis dementia  populations: Protocol for a systematic review
Source: HRB Open Res. 2025 Sep 29;8:11. Originally published 2025 Jan 23. [Version 3] doi: 10.12688/hrbopenres.14064.3 (PMC11950719; doi:10.12688/hrbopenres.14064.3)
Supplement: Peer review history

# Reviewer report: Reviewer response for version 2

**DOI:** 10.21956/hrbopenres.15492.r46287

**Published:** 2025-03-27

**Authors:** Amritpal Rehill

**Affiliations:** 1 London School of Economics and Political Science, London, UK

The authors have done a good job in producing a well written and well considered protocol. They have convincingly justified why it is important to look at the pre-diagnosis dementia population to maximise the potential effect of intervening. The methods are described in detail, including what databases will be searched, what criteria for inclusion/exclusion will be used, what data will be extracted and how results will be presented.  I would be interested in reading the resulting review. I have a few comment/suggestions, as follows:

- It might be a good idea to briefly discuss how accurately we can currently predict who may or may not go on to have dementia in the future. It was mentioned that pharmacological interventions in the pre-diagnosis context were to be considered- presumably it would be preferred to go ahead with this for those we are particularly confident will go on to be diagnosed with dementia.
- Is there a cut-off in terms of how old a study can be to be included in the review? Tied to this, for older studies, something that might be considered is that costs for different components of the interventions may be different now compared to in the past (especially if technology was involved, or if specific drugs are no longer under patent). It might be that cost-effectiveness of some interventions may be different if the intervention were provided today, however without being able to repeat the original study this might need to be listed as a limitation.
- Tied to the above, under study details extracted, it may be useful for the year(s) in which the intervention was administered to be extracted.  
- On similar lines to time, place may also play a factor in determining cost-effectiveness. As an example, for a given intervention, studies from the US and UK may suggest it is cost-effective, but there might not be studies from other countries and there might be reasons so suggest cost-effectiveness might not hold true in other settings (e.g. in developing countries), where costs may differ. I was wondering if the authors were planning to evaluate/comment on the generalisability of the resulting cost-effectiveness estimates.   
- Alongside evaluating cost-effectiveness, I was wondering if there were any plans to discuss potential differences regarding implementation. For example, a group intervention studied in the US/UK may have been found to be cost-effective, but for those living in a different country where there is more stigma around dementia, individuals may not want to attend group sessions to receive the intervention. I appreciate that this is not the core focus of the study, but I think providing a brief description of this to speak to the generalisability of results where studies exist only in a few similar countries/settings would help to contextualise the findings.
- It is fantastic to see that the focus is not only on cost-effectiveness but also on methods and methods used to calculate costs. Currently there is no consensus on how best to value hours of unpaid care (e.g. replacement cost approach or opportunity cost approach), and this decision can have a large impact on the ultimate cost-effectiveness calculation. This alongside other methodological decisions being noted/tracked/compared should prove useful.    

Is the study design appropriate for the research question?

Yes

Is the rationale for, and objectives of, the study clearly described?

Yes

Are sufficient details of the methods provided to allow replication by others?

Yes

Are the datasets clearly presented in a useable and accessible format?

Not applicable

Reviewer Expertise:

I am a quantitative researcher in the area of social care and have worked on numerous projects involving people with dementia. This has included multiple economic evaluations of trials for non-pharmacological interventions for people with dementia, as well as projecting costs and outcomes for both people with dementia and their carers.

I confirm that I have read this submission and believe that I have an appropriate level of expertise to confirm that it is of an acceptable scientific standard.

# Response

**Published:** 2025-09-26

**Authors:** Men Hoang

**Affiliations:** Trinity College Dublin School of Medicine, Dublin, Leinster, Ireland

**1. It might be a good idea to briefly discuss how accurately we can currently predict who may or may not go on to have dementia in the future. It was mentioned that pharmacological interventions in the pre-diagnosis context were to be considered- presumably it would be preferred to go ahead with this for those we are particularly confident will go on to be diagnosed with dementia.**

**Response:** We appreciate your review of our protocol and the insightful comments you provided. We will think about this point in our review and discuss its implications for identifying appropriate target populations.

**2. Is there a cut-off in terms of how old a study can be to be included in the review?**

**Response: **Thank you for your thoughtful comment. We include all publications from January 1, 2000, to January 14, 2025.

 We added a sentence in the protocol:

 “We searched for articles published between January 1, 2000, and January 14, 2025.”

**Tied to this, for older studies, something that might be considered is that costs for different components of the interventions may be different now compared to in the past (especially if technology was involved, or if specific drugs are no longer under patent). It might be that cost-effectiveness of some interventions may be different if the intervention were provided today, however without being able to repeat the original study this might need to be listed as a limitation.**

**Response:** In our review:

 1) We adjust cost of intervention up to 2024 value and convert to int$ 

 2) We will consider mentioning this limitation of simple adjustments in discussing the final paper. 

**3. Tied to the above, under study details extracted, it may be useful for the year(s) in which the intervention was administered to be extracted.**

**Response:** Thank you for your comment. In our review, we extract the year of publication and price year when costs were reported (if available).

**4. On similar lines to time, place may also play a factor in determining cost-effectiveness. As an example, for a given intervention, studies from the US and UK may suggest it is cost-effective, but there might not be studies from other countries and there might be reasons so suggest cost-effectiveness might not hold true in other settings (e.g. in developing countries), where costs may differ.**

**Response: 4.** Thank you for your suggestion. Indeed, we will have a paragraph to discuss country-specific decision-making in considering the cost-effectiveness of interventions in our review manuscript based on the final extracted data.

**I was wondering if the authors were planning to evaluate/comment on the generalisability of the resulting cost-effectiveness estimates**

**Response:** Two willingness-to-pay (WTP) benchmarks were used to demonstrate the comparative cost-effectiveness of the strategies: $150,000 per QALY, indicative of the relatively unregulated market context in the United States (US) 18, and £12,936 per QALY, representing the more resource-limited system in the United Kingdom (UK)19. These values were selected to capture the range between higher and lower reference points. The cost-effectiveness plane is used solely for illustrative purposes, we will not alter the original conclusion regarding the cost-effectiveness of the included articles.

**5. Alongside evaluating cost-effectiveness, I was wondering if there were any plans to discuss potential differences regarding implementation.**

**For example, a group intervention studied in the US/UK may have been found to be cost-effective, but for those living in a different country where there is more stigma around dementia, individuals may not want to attend group sessions to receive the intervention.**

**I appreciate that this is not the core focus of the study, but I think providing a brief description of this to speak to the generalisability of results where studies exist only in a few similar countries/settings would help to contextualise the findings.**

**Response:** Thank you for your thoughtful comment. We will take your suggestion into account in the discussion section of our review. Exploration of whether included studies were actually implemented is a very good suggestion but is unfortunately beyond the scope at this time. In the final paper, we will discuss this as a recommendation from this review. The point on generalisability is well taken and, at this protocol paper stage (for the benefit of non-economists.

**6. It is fantastic to see that the focus is not only on cost-effectiveness but also on methods and methods used to calculate costs. Currently there is no consensus on how best to value hours of unpaid care (e.g. replacement cost approach or opportunity cost approach), and this decision can have a large impact on the ultimate cost-effectiveness calculation. This alongside other methodological decisions being noted/tracked/compared should prove useful.**

**Response: **Thank you for your comment. For each included article, we will summarise its details, such as: title, purpose, country, funding source, type of EE, study design, type of model (if applicable), time horizon, perspective, population, intervention, comparator, resource measurement, utility measurement, uncertainty analysis, currency and price year, discount, quality of article in terms of our newly developed framework to provide an objecty score from CHEERS checklist, economic measure in terms of total cost, total QALY, incremental cost and QALY, intervention cost.

 We wholeheartedly agree with this limitation in the literature and, informed by and conducted in parallel to this review, we are conducting work to reach greater consensus (Trepel, D. (2019). Core outcome set for economic evaluation of dementia interventions. COMET Initiative. Retrieved April 7, 2025, from https://www.comet-initiative.org/Studies/Details/1737). This review, along with reviews of post diagnostic EEs (Scanlon, H., Saha, S., Trepel, D. (2024). Economic evaluations of interventions for people living with dementia and their caregivers: A systematic review and core outcome set of resource use. ResearchGate), are extracting data on varying resource use data, and this will inform a consensus exercise to ultimately produce a core outcomes set for future dementia economic evaluation. To extend this discussion, we will also extract which unit costs are applied to informal care across EEs.

# Reviewer report: Reviewer response for version 2

**DOI:** 10.21956/hrbopenres.15492.r46288

**Published:** 2025-03-22

**Authors:** Jiaer Lin

**Affiliations:** 1 The Chinese University of Hong Kong (CUHK), Hong Kong, Hong Kong

**There are some points are not clear and please consider to clarify:**

**Introduction:**

 1. Is there any definition for pre-diagnosis stage in either clinical practice or research?

 2. What’s the differences with MCI? If prior research have already reviewed those MCI, what value of this research will be contributed to?

**Methods:**

**Eligibility**

 1. For the target population, how to identify if the articles includes individuals at any stage prior dementia diagnosis? By what definition or any tests (e.g., MMSE) used? If by tests, what results are considered to be eligible? 

 2. Intervention: Would it be possible that (3) non-pharmacotherapy have already included (4) management strategies at home, in the community, and in long-term care?

 3. Outcomes: Please specify what perspective the authors decided to adopt when reporting the cost outcomes, will the authors include all cost items? Or only for one perspective? If for all, how about costs that published in separate articles?

 4. Study design:

 - Trail-based and model-based EEs are very different study types. For model-based, additional information such as modeling technique, parameters, etc. 

 are important and should also be considered and specified clearly.

 - Will the authors consider include multiple (≥2) articles that published for the same intervention for different outcomes, say cost outcomes in one article and effectiveness such as clinical outcomes in anthter.

**Data synthesis**

 1. There are three guidelines that provides instructions of

 conducting systematic review on cost-effectiveness studies (Cochrane HandBook, CRD from York,  and ISPOR), the authors should consider to follow one of each for the data synthesis process.

 2. The authors stated that "Where studies report both total costs and outcomes expressed in QALYs, results will be adjusted and will be visually presented on a cost-effectiveness plane to facilitate decision-making." The authors should clarify more details for the following points;

 - How to adjust the results?

 - How about costs in different countries? Any standardized method will be used?

 - What threshold of williness-to-pay will the authors use to present the cost-effectiveness plane? 

 - Will this study conclude with which strategy is cost-effective/cost-saving or not, how?

 - There are others details need to consider especially for model-based EEs. The authors could reconsider whether to include model-based studies or not.

**Quality appraisal**

 In addition to the methodological quality of the EEs, the authors should also evaluate the publication bias of the included studies, following the Cochrane guideline. The authors can take our prior systematic review as reference for the methodology.

Is the study design appropriate for the research question?

Yes

Is the rationale for, and objectives of, the study clearly described?

Yes

Are sufficient details of the methods provided to allow replication by others?

Partly

Are the datasets clearly presented in a useable and accessible format?

Yes

Reviewer Expertise:

I am a health economist and have been published work on systematic reviews related to dementia care and integrated care.

I confirm that I have read this submission and believe that I have an appropriate level of expertise to confirm that it is of an acceptable scientific standard, however I have significant reservations, as outlined above.

## References

[1] : Supporting Informal Caregivers of People With Dementia in Cost-Effective Ways: A Systematic Review and Meta-Analysis. *Value Health* .2021;24(12) : 10.1016/j.jval.2021.05.011 1853-1862 10.1016/j.jval.2021.05.011 34838284

[2] : Cost‐effectiveness of non‐pharmacological interventions for patients with dementia: A systematic review and meta‐analysis. *Alzheimer's & Dementia* .2021;17(S10) : 10.1002/alz.053962 10.1002/alz.053962

[3] : Cost-effectiveness of pharmacological therapies for people with Alzheimer's disease and other dementias: a systematic review and meta-analysis. *Cost Eff Resour Alloc* .2022;20(1) : 10.1186/s12962-022-00354-3 19 10.1186/s12962-022-00354-3 35443684 PMC9022294

# Response

**Published:** 2025-09-26

**Authors:** Men Hoang

**Affiliations:** Trinity College Dublin School of Medicine, Dublin, Leinster, Ireland

Thank you for taking the time to review our protocol.

**Introduction:**

**1. Is there any definition for pre-diagnosis stage in either clinical practice or research?**

**Response:** Thank you for your comments.

 In the literature, we found this paper defined “"Pre-diagnosis was defined as the period from the initial onset of symptoms up to the formal diagnosis”. ( https://doi.org/10.1017/S1041610217000862)

 In our review, we added a sentence to clarify the definition of the pre-diagnosis dementia population as “individuals who have not yet been formally diagnosed”.

 “Thus, it is essential to identify and implement cost-effective strategies targeting individuals who have not yet been formally diagnosed (i.e., those in the pre-diagnosis dementia populations) to help reduce the rising impact of dementia”.

**2. What’s the differences with MCI? If prior research have already reviewed those MCI, what value of this research will be contributed to?**

**Response: **In our review, pre-diagnosis dementia populations are broader than MCI.

 In the introduction, we added a sentence for clarification. “However, a comprehensive review encompassing all strategies specifically targeting pre-diagnosis dementia populations, which include the general population, at-risk groups, and individuals with MCI without detectable AD pathology, is lacking in the literature.”

**Methods:**

**Eligibility**

**1. For the target population, how to identify if the articles includes individuals at any stage prior dementia diagnosis? By what definition or any tests (e.g., MMSE) used? If by tests, what results are considered to be eligible?**

**Response: **1. We identified the target population according to how the authors described the target populations in their economic evaluations. We added following sentence in our protocol:

 “The pre-diagnosis dementia population is typically defined by authors who explicitly state that their studies start with a cohort described as having “no dementia,” or as comprising individuals with mild cognitive impairment (MCI) or at-risk population.”

**2. Intervention: Would it be possible that (3) non-pharmacotherapy have already included (4) management strategies at home, in the community, and in long-term care?**

**Response**: We revised the protocol and categorised interventions into three groups: identification, risk reduction, and others. The latter group will compass studies that do not fall into either identification or risk reduction.

**3. Outcomes: Please specify what perspective the authors decided to adopt when reporting the cost outcomes, will the authors include all cost items? Or only for one perspective? If for all, how about costs that published in separate articles?**

**Response: ** We do not adopt any perspective whilst critically appraising the literature. But rather, we "document stated perspectives" . We extracted the average total cost and intervention cost (if reported) as they may (or may not) relate to stated perspectives.

**4. Study design:**

**- Trail-based and model-based EEs are very different study types. For model-based, additional information such as modeling technique, parameters, etc.**

**are important and should also be considered and specified clearly.**

**Response:** We include articles as long as they are considered full economic evaluations according to Drummond’s classification scheme. This means we include both trial-based and model-based full economic evaluations.

**- Will the authors consider include multiple (≥2) articles that published for the same intervention for different outcomes, say cost outcomes in one article and effectiveness such as clinical outcomes in anthter.**

**Response**: The data extraction stage is a memoryless process and no attempt is made to cross reference studies to check if multiple articles were published from the same study of a single intervention. However, at analysis, where there is sufficient evidence of duplication of reporting the same findings, the study would be flagged as such.

**Data synthesis**

**1. There are three guidelines that provides instructions of conducting systematic review on cost-effectiveness studies (Cochrane HandBook, CRD from York,  and ISPOR), the authors should consider to follow one of each for the data synthesis process.**

**Response: **Thank you for your suggestion. All systematic review processes aim to adhere to standards set out in the Cochrane Handbook and specifically Chapter 20: Economic evidence.

 The results are reported in accordance with the Preferred Reporting Items for Systematic Reviews and Meta-Analyses (PRISMA) guidelines.

 The template for summarising data extracted from each individual study was based on the former template of CRD NHS EED database.

**2. The authors stated that "Where studies report both total costs and outcomes expressed in QALYs, results will be adjusted and will be visually presented on a cost-effectiveness plane to facilitate decision-making." The authors should clarify more details for the following points;**

**- How to adjust the results?**

**- How about costs in different countries? Any standardized method will be used?**

**Response:** Thanks to your comment, we identify there is an error in our statement (i.e it should be incremental cost not total cost).The cost-effectiveness plane is used solely for illustrative purposes but adjustments are required:We used CPI and PPP method, converted cost to international dollar in 2024 prices. We added in the protocol as:“the incremental cost will be converted to international dollars (value in 2024) using the purchasing power parity from the International Monetary Fund dataset ^17^, and visually presented on a cost-effectiveness plane to facilitate comparison.”

**- What threshold of williness-to-pay will the authors use to present the cost-effectiveness plane?**

**Response**: Two willingness-to-pay (WTP) benchmarks were used to demonstrate the comparative cost-effectiveness of the strategies: $150,000 per QALY, indicative of the relatively unregulated market context in the United States (US) 18, and £12,936 per QALY, representing the more resource-limited system in the United Kingdom (UK)19. These values were selected to capture the range between higher and lower reference points.

**- Will this study conclude with which strategy is cost-effective/cost-saving or not, how?**

**Response: **Based on the CE-plane, we can see if the intervention is in the dominant quadrant. The cost-effectiveness plane is used solely for illustrative purposes, we will not alter the original conclusion regarding the cost-effectiveness of the included articles. The intention of this review is not to determine whether any given strategy is cost effective or not (because, as the reviewer indicates, different countries will have different willingness to pay threshold). Rather, the intention is to systematically identify and critical appraisal all relevant studies. By extension, the illustration on the cost effectiveness plane provide a synthesis for comparison of the relative efficiency of each approach, however, this is not intended to endorse use in any jurisdiction but rather encourage countries to conduct their own country level appraisal (in which they may opt to adapt analyses highlighted by our review).

**- There are others details need to consider especially for model-based EEs. The authors could reconsider whether to include model-based studies or not?**

**Response:** The article is intended to provide a compendium of all EEs for non-economist audience and, as part of the wider work, we wish this to help readers understand there are two main sources of cost-effectiveness evidence.

**Quality appraisal**

**In addition to the methodological quality of the EEs, the authors should also evaluate the publication bias of the included studies, following the Cochrane guideline. The authors can take our prior systematic review as reference for the methodology.**

**Response:** Thank you for your advice. In EE, we do not have publication bias estimation. But in many reviews, they estimate the publication  bias of the included intervention. We are not doing that in this review.

 Once again, thank you very much for providing comments. We believe that your comments will help us in improving the clarification and transparency of this protocol

# Reviewer report: Reviewer response for version 2

**DOI:** 10.21956/hrbopenres.15492.r46271

**Published:** 2025-03-05

**Authors:** Joseph Kane

**Affiliations:** 1 Queen's University Belfast, Belfast, Ireland

I thank the authors for being so receptive to my comments. My queries have been addressed in this revised manuscript. I look forward to seeing the results of this study.

Is the study design appropriate for the research question?

Partly

Is the rationale for, and objectives of, the study clearly described?

Yes

Are sufficient details of the methods provided to allow replication by others?

Yes

Are the datasets clearly presented in a useable and accessible format?

Not applicable

Reviewer Expertise:

I am a dementia clinician and researcher. I have limited knowledge of economic methodologies but do have experience in systematic review.

I confirm that I have read this submission and believe that I have an appropriate level of expertise to confirm that it is of an acceptable scientific standard.

# Response

**Published:** 2025-09-26

**Authors:** Men Hoang

**Affiliations:** Trinity College Dublin School of Medicine, Dublin, Leinster, Ireland

Thank you, Dr. Kane, for your comments; they definitely help us enhance the clarity of our protocol.

# Reviewer report: Reviewer response for version 1

**DOI:** 10.21956/hrbopenres.15446.r45165

**Published:** 2025-02-10

**Authors:** Joseph Kane

**Affiliations:** 1 Queen's University Belfast, Belfast, Ireland

Thank you for the opportunity to review this important work.

 The first paragraph refers to dementia as single condition when it more accurately represents a construct by which a series of neurodegenerative processes manifest. Consider "Dementia is a group of conditions characterised …"

 The second paragraph includes a sentence suggesting that "screening tests that enable individuals to maintain independent living" doesn't quite make - it seems like an intervention rather than a test is more likely to maintain independence.

 I do follow the pragmatic approach to excluding populations with MMSE <24 but I would dispute that such populations are commonly predefined this way, certainly not clinically or in clinical trials.

 I don't quite follow the exclusion of those with MCI-AD and feel this should be better and more explicitly justified.  

 This protocol would otherwise appear well constructed and justifiable.

Is the study design appropriate for the research question?

Partly

Is the rationale for, and objectives of, the study clearly described?

Yes

Are sufficient details of the methods provided to allow replication by others?

Yes

Are the datasets clearly presented in a useable and accessible format?

Not applicable

Reviewer Expertise:

I am a dementia clinician and researcher. I have limited knowledge of economic methodologies but do have experience in systematic review.

I confirm that I have read this submission and believe that I have an appropriate level of expertise to confirm that it is of an acceptable scientific standard, however I have significant reservations, as outlined above.

# Response

**Published:** 2025-02-24

**Authors:** Men Hoang

**Affiliations:** Trinity College Dublin School of Medicine, Dublin, Leinster, Ireland

Thank you, Dr. Kane, for reviewing our protocol and providing us with valuable comments.

**Reviewer's comment 1:** "The first paragraph refers to dementia as single condition when it more accurately represents a construct by which a series of neurodegenerative processes manifest. Consider "Dementia is a group of conditions characterised …"

**Response:** Thank you for pointing it out. We revised the first sentence accordingly.

 "Dementia is a group of conditions characterised by deterioration of multiple cognitive domains that significantly affects daily functioning”.

**Reviewer's comment 2: **The second paragraph includes a sentence suggesting that "screening tests that enable individuals to maintain independent living" doesn't quite make - it seems like an intervention rather than a test is more likely to maintain independence.

**Response: **Thanks for your comment. We revised as following:

 “Additionally, given the significant costs associated with dementia, early interventions, such as identification strategies, may enable timely diagnosis and treatment, helping to mitigate complications, support independent living, and give family members more time to prepare.”

**Reviewer's comment 3:** I do follow the pragmatic approach to excluding populations with MMSE <24 but I would dispute that such populations are commonly predefined this way, certainly not clinically or in clinical trials.

**Response:** Thank you for your comment. Initially, we used MMSE scores to predefine the included populations. However, after the screening process, we realised that defining the pre-diagnosis population based on MMSE scores is not a common approach. The population is now defined as follows:

 “Population: The target population includes individuals at any stage prior to having a formal diagnosis of dementia, such as the general population, people at risk of dementia, those with mild cognitive impairment (MCI), caregivers, and patient-caregiver dyads.”

**Reviewer's comment 4: **I don't quite follow the exclusion of those with MCI-AD and feel this should be better and more explicitly justified. 

**Response: **To confirm that MCI is due to AD, biomarkers reflecting amyloid and tau pathology (core features of Alzheimer’s) are assessed. Thus, we consider MCI due to AD is post-diagnosis population.

 We added:

 “This is because the criteria for MCI due to AD must be confirmed by biomarkers to determine the likelihood that the syndrome results from the underlying pathophysiology of AD ^16^.

**Reviewer's comment 5: **This protocol would otherwise appear well constructed and justifiable.

 Response: Thank you.
